# Mesenchymal Stromal Cell-Based Immunotherapy for Type 1 Diabetes: From Bench to Bedside

**DOI:** 10.3390/ijms272110005

**Published:** 2026-11-08

**Authors:** Alexey Lupatov, Irina Pokusaeva, Irina Kholodenko, Marina Shestakova, Kirill Larin, Rita Khusainova, Ildar Minniakhmetov, Vladimir Chekhonin, Konstantin Yarygin, Natalia Mokrysheva

**Affiliations:** 1 Institute of Biomedical Chemistry (IBMC), 119121 Moscow, Russia; pokusaeva.i@yandex.ru (I.P.); irkhol@yandex.ru (I.K.); kir.lar2001@gmail.com (K.L.); kyarygin@yandex.ru (K.Y.);; 2 Endocrinology Research Centre, 117292 Moscow, Russia; shestakova.marina@endocrincentr.ru (M.S.); khusainova.rita@endocrincentr.ru (R.K.); minniakhmetov.ildar@endocrincentr.ru (I.M.); chekhoninnew@yandex.ru (V.C.); mokrisheva.natalia@endocrincentr.ru (N.M.)

**Keywords:** type 1 diabetes mellitus, mesenchymal stromal cells, autoimmunity, cell-based therapy, immunomodulation

## Abstract

Type 1 diabetes mellitus (T1D) is an autoimmune disease characterized by the progressive destruction of pancreatic β-cells. Despite advances in glucose monitoring and insulin delivery, current treatments do not directly target the underlying autoimmune process. The administration of mesenchymal stromal cells (MSCs) represents a promising therapeutic approach due to their immunomodulatory, anti-inflammatory, and regenerative properties. Unlike therapies targeting individual immune pathways, MSCs exert broad immunomodulatory effects. This review integrates current knowledge of T1D immunopathogenesis with evidence on MSC-mediated immunomodulation, highlighting the ability of MSCs to modulate multiple interconnected mechanisms, including innate and adaptive immunity, antigen presentation, inflammatory signaling, and immune tolerance. Preclinical evidence suggests that MSCs may attenuate inflammation, regulate immune responses, preserve β-cell function, and improve metabolic outcomes. A preliminary but encouraging finding from clinical studies is that MSC administration may produce sustained beneficial effects after a limited number of injections. However, this observation remains tentative and requires confirmation in further studies. Standardized treatment protocols, adequately powered controlled trials, and long-term follow-up are needed to establish the clinical efficacy, durability of response, and optimal use of MSC-based therapy in T1D.

## 1. Introduction

Type 1 diabetes mellitus remains a leading global medical and societal challenge, characterized by the early disease onset, lifelong dependence on exogenous insulin therapy, rigorous follow-up requirements, and substantial healthcare expenditure [1,2]. Insulin replacement therapy, currently the only established means of sustaining life in patients with type 1 diabetes (T1D), is not without limitations [3]. Despite considerable advances in continuous glucose monitoring systems and insulin pump technology, exogenous insulin cannot replicate the fine, dynamic regulation of glucose metabolism ensured by healthy β-cells [4]. As a result, patients experience persistent blood glucose fluctuations that substantially increase the risk of severe hypoglycemia or hyperglycemia—which not only impair quality of life but also drive higher rates of cardiovascular complications and overall mortality [5]. The main limitation of T1D therapy is that insulin administration remains purely substitutive: it compensates for hormone deficiency but has no effect on T1D immunopathogenesis. Consequently, autoimmune attack continues to progress unabated, gradually eliminating residual pancreatic β-cells even under optimally adjusted insulin regimens [3,6,7]. Given this, the search for novel T1D therapies modulating the immune response and preserving islet function is critically important [8].

In recent years, personalized cell-based approaches, including tolerogenic dendritic cells (tolDCs) and adoptive therapy with autologous regulatory T cells (Tregs), have demonstrated some efficacy [9,10,11,12,13] in T1D treatment. Unlike targeted therapies aimed at a single immunological mechanism (e.g., anti-CD3 or anti-CD20 antibodies) [14,15], cell-based approaches are designed to exert a more comprehensive effect [16,17]. However, these therapies target only a limited number of nodes of the immune cascade, whereas the autoimmune process in T1D constitutes a complex network characterized by high plasticity, cross-reactivity, and compensatory mechanisms.

Mesenchymal stromal cell-based therapy has a pleiotropic effect on the recipient’s immune system [18]. Unlike narrowly targeted agents, mesenchymal stromal cells (MSCs) possess an intrinsic capacity to modulate a broad range of immune processes: they induce functional anergy in autoreactive T lymphocytes and promote expansion of Tregs [19]; suppress B-cell proliferation and the production of autoantibodies [20]; block dendritic cell (DC) maturation; and shift the macrophage phenotype from a pro-inflammatory (M1) to an anti-inflammatory (M2) profile [21], while simultaneously normalizing the systemic cytokine milieu. A key feature is that this immunomodulation appears to occur without inducing systemic immunosuppression or cytotoxicity, thereby preserving host immunity against infections and maintaining immune surveillance [22]. The clinical safety and therapeutic efficacy of various MSC types (bone marrow-derived, adipose-derived, umbilical cord-derived, and others) have been supported by randomized trials across a range of severe autoimmune and immune-mediated conditions. An important advantage of MSCs is their low immunogenicity, which allows the use of both autologous and allogeneic cells in therapy. Specifically, allogeneic MSCs have shown marked efficacy in treating acute graft-versus-host disease [23], have been associated with remission in refractory systemic lupus erythematosus [24], and may slow neuroinflammation in progressive forms of multiple sclerosis [25].

The complexity of T1D immunopathogenesis and the ability of MSCs to simultaneously target multiple steps of the immune cascade make them a promising candidate for disease-modifying therapy. The unique combination of their broad immunomodulatory profile, robust paracrine activity, and favorable safety record provides a strong scientific rationale for actively exploring MSCs to induce durable immune tolerance and preserve long-term β-cell function in T1D [26,27].

The aim of this review is to analyze and synthesize the available evidence on the therapeutic potential of MSCs in T1D. Because autoimmune β-cell destruction drives T1D, particular emphasis is placed on the immunomodulatory properties of MSCs that allow them to target key nodes of immunopathogenesis, as well as on preclinical and clinical findings that either support or challenge this approach. A separate aspect of the analysis concerns the impact of the hyperglycemic diabetic microenvironment on the functional activity of transplanted MSCs, a factor that determines the real-world effectiveness of cell-based therapy under disease conditions. Finally, this review broadens the perspective beyond islet preservation by assessing the ability of MSCs to modulate the systemic autoimmune inflammation that underlies long-term complications—diabetic neuropathy, nephropathy, and retinopathy—which remain clinically relevant even after complete loss of pancreatic endocrine function. Collectively, the work aims to provide a comprehensive picture of where MSC therapy stands in the current treatment paradigm for T1D: from pathophysiological rationale to unresolved challenges and prospects for clinical translation.

## 2. Epidemiology and Global Burden of T1D

Epidemiological data point to marked geographic heterogeneity and a steady global rise in the incidence of T1D [28]. Current estimates from a systematic review and meta-analysis indicate that T1D accounts for roughly 9.5% of all diabetes cases worldwide, with annual incidence ranging from 0.5 to 40 new cases per 100,000 persons depending on the region [29]. Historically, the highest incidence rates have been reported in Finland, Sardinia (Italy), and Sweden, where they exceed 30–40 cases per 100,000 children per year, whereas in East Asian countries (Japan, China) rates remain below 5 per 100,000 [4]. Between 1989 and 2003, the average annual increase in incidence among European children aged 0–14 years was 3.9%, with the most pronounced rise occurring in the 0–4 year age group [30]. A subsequent systematic review confirmed a similar upward trend in young adults: in Scandinavia, the United Kingdom, and Canada, T1D incidence in individuals 15–40 years of age rose by 2–5% annually, indicating that the disease is extending beyond the pediatric age range [31]. Although traditionally viewed as a disorder primarily affecting children and adolescents, the past few decades have witnessed a marked rise in the frequency of adult-onset cases. Consequently, efforts to refine clinical management and advance pathogenesis-oriented therapeutic strategies must remain inclusive of all age groups [32].

Unfortunately, the previously projected rise in T1D incidence has not only materialized but has also taken on new demographic features. According to current global estimates for 2025, approximately 9.5 million people worldwide are living with T1D, of whom 1.85 million are children and adolescents under 20 years of age [33]. Whereas past decades placed major emphasis on the explosive increase in incidence among young children, more recent studies document a sustained global rise in incidence among adolescents as well [34]. Moreover, the paradigm of T1D as an exclusively “juvenile” disease has now been definitively revised: current epidemiological models indicate that 64% of all new T1D cases are diagnosed in individuals aged 20 years and older [35]. In developed countries, including the United States, T1D prevalence among adults now exceeds that among children, with approximately 5 cases per 1000 adults versus 4 cases per 1000 children and adolescents as of 2019–2022 [36]. Mathematical modeling of current trends suggests that if the observed rates of incidence growth persist, the total number of T1D cases in the population could increase by roughly 65% over the coming decades [37].

The data presented support the hypothesis that environmental, ethnic, and socioeconomic factors exert a substantial influence on the course of the autoimmune process, which is consistent with findings from international studies documenting regional differences in disease incidence [38]. Genetic background currently appears to play a less decisive role in disease development than it did in the past. Epidemiological evidence suggests that environmental triggers are increasingly initiating the autoimmune process in individuals with modest or low genetic susceptibility, positioning the immune system as a key modifiable target for therapeutic intervention [39,40]. An important positive trend is the increased life expectancy of patients with T1D: the median disease duration from onset to death rose from 15.4 to 19.9 years over the observation period, while the mean age at death reached 52.7 years [41]. Nevertheless, glycemic control targets remain inadequately met, with only 34.7% of T1D patients achieving an HbA1c level below 7.0%—underscoring the pressing need for disease-modifying therapies capable not only of replacing insulin but also of suppressing the autoimmune process [42].

## 3. Immunopathogenesis of T1D and Therapeutic Targets for Cell-Based Approaches

### 3.1. Genetic Susceptibility and Triggering Factors

The development of T1D is driven by a complex interplay between genetic predisposition and environmental triggers, which jointly disrupt the mechanisms of immune tolerance. To date, more than 40 genetic loci influencing T1D susceptibility have been identified, with approximately 40–50% of the inherited risk associated specifically with human leukocyte antigen (HLA) genes, which determine the presentation of autoantigens to the immune system [43]. The highest risk is conferred by the haplotypes DRB103:01-DQA105:01-DQB102:01 and DRB104:05-DQA103:01-DQB103:02, named in serological nomenclature as DR3-DQ2 and DR4-DQ8, respectively. These haplotypes associated with Major Histocompatibility Complex (MHC) class II molecules with a specific peptide-binding groove structure that presents islet autoantigens (such as insulin, Glutamic Acid Decarboxylase 65 (GAD65), Insulinoma-associated protein 2 (IA-2), and Zinc Transporter 8 (ZnT8) to autoreactive CD4^+^ T lymphocytes [44]. Heterozygosity for DR3/DR4 multiplicatively increases the risk of clinical disease onset, whereas the HLA-DQB1*06:02 allele exerts a pronounced protective effect by promoting robust central tolerance in the thymus [45].

Beyond the HLA region, genome-wide association studies have identified more than 60 non-HLA loci that collectively explain up to 15% of the genetic risk, with polymorphisms in the *PTPN22*, *CTLA4*, and *IL2RA* genes playing a key role [46,47]. The *PTPN22* R620W variant alters the activity of the LYP tyrosine phosphatase, leading to a lowered T-cell receptor activation threshold and allowing autoreactive clones to escape negative selection [48]. Certain *CTLA4* polymorphisms weaken the inhibitory signal required for the induction of T-lymphocyte anergy and the functional activity of Tregs [49]. Variations in the *IL2RA* gene can disrupt the expression of the interleukin-2 receptor α-chain, critically impairing the survival, proliferation, and suppressive function of Tregs that maintain peripheral tolerance [50].

**Figure 1:**
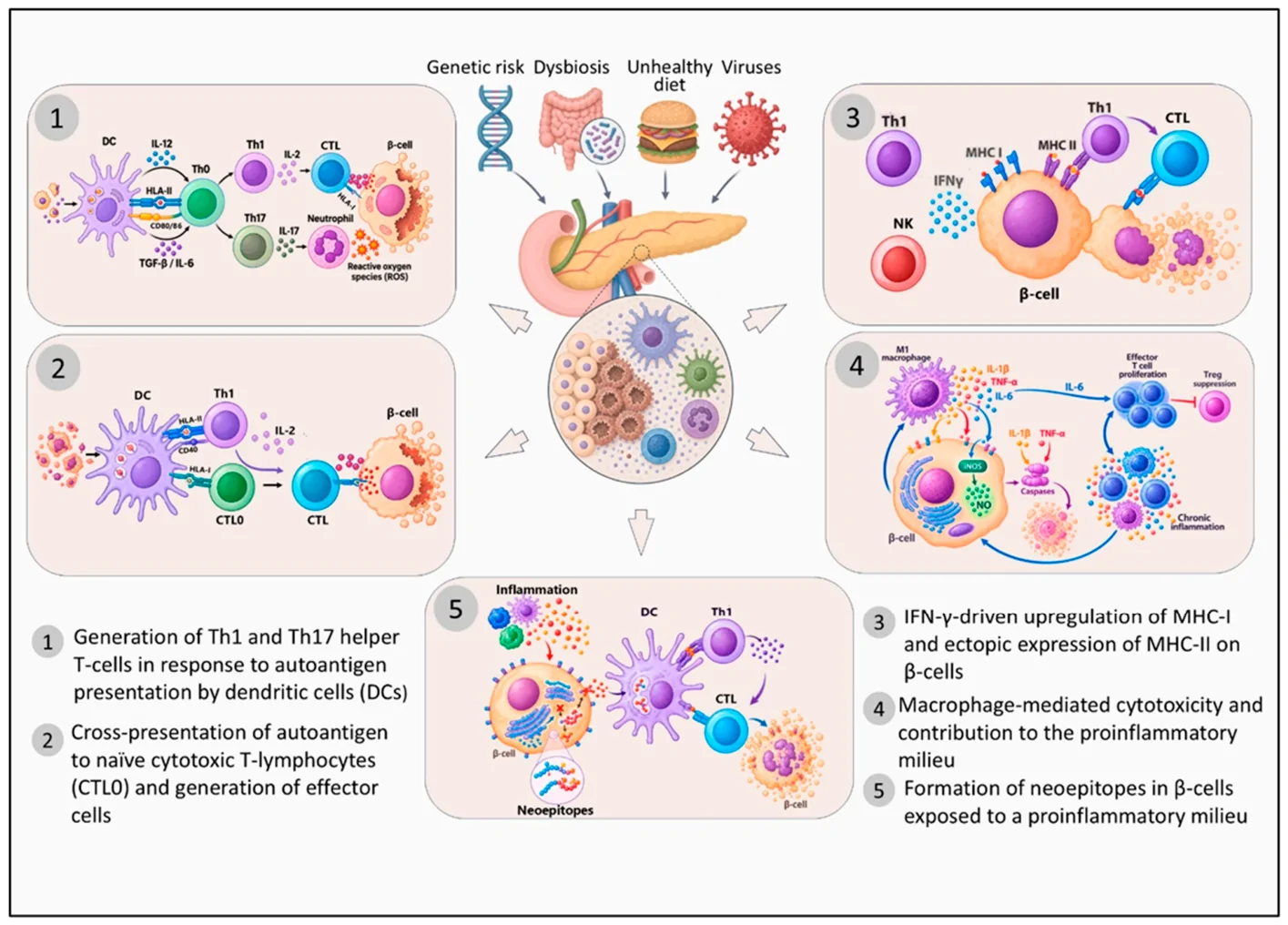
Key immunological mechanisms in the pathogenesis of T1D. (1) DCs maturing in a proinflammatory microenvironment can process fragments of β-cells killed by exogenous triggers or autoimmune attack and present their epitopes to naïve T-helper (Th0). Depending on DC-secreted cytokines, Th0 may differentiate into T-helper 1 (Th1) or T-helper 17 (Th17) subsets. Th1 promote cytotoxic T lymphocyte (CTL) activity against β-cells, whereas Th17 recruit and activate neutrophils, which destroy islet tissue via reactive oxygen species (ROS) release. (2) CD40-dependent interaction with Th1 activates DCs to cross-present autoepitopes on HLA-I to naïve CTLs, which then differentiate into effector CTLs and destroy β-cells. (3) IFN-γ from activated Th1 and natural killer (NK) cells induces normally absent HLA-II on β-cells and upregulates HLA-I, collectively enhancing adaptive immunity to autoantigens. (4) M1 macrophage-derived cytokines, including interleukin-6 (IL-6), directly activate inducible nitric oxide synthase (iNOS) and caspases in β-cells, inducing apoptosis, and sustain chronic inflammation, effector T-cell proliferation, and Treg suppression. Chronic inflammation, in turn, reinforces the M1 phenotype. (5) Under inflammatory conditions, post-translational modification of certain proteins may be dysregulated in β-cells. The resulting neoepitopes can be presented by DCs to lymphocytes that have passed central tolerance selection. Neoepitope-specific effector CTLs can then recognize and eliminate β-cells presenting these neoepitopes.

However, carrying predisposition alleles alone is insufficient for disease manifestation. The autoimmune process is triggered by environmental factors acting during early-life immune system development. Viral infections, primarily enteroviruses including Coxsackievirus B, are considered the most likely triggers, initiating pathogenesis through mechanisms of molecular mimicry, immune cell activation at sites of inflammation, and direct viral destruction of β-cells [51,52]. Gut microbiota dysbiosis, characterized by reduced microbial diversity, a deficit of short-chain fatty acid-producing bacteria, and impaired intestinal barrier permeability, leads to systemic pro-inflammatory activation and disrupted differentiation of regulatory lymphocytes [53,54]. Dietary factors also contribute significantly: the early introduction of cow’s milk and gluten, along with deficiencies in vitamin D and omega-3 polyunsaturated fatty acids, correlate with an increased risk of developing autoantibodies against pancreatic islet cells. This has been confirmed by large-scale prospective studies tracking children with genetic risk from birth to clinical onset [55,56]. The cumulative effect of these triggers in a genetically predisposed individual disrupts the balance between the effector and regulatory arms of the immune system, creating the conditions for the initiation of irreversible β-cell destruction (Figure 1).

### 3.2. Impaired Immune Tolerance and Activation of Autoreactive Clones

The development of autoimmune attack in T1D begins with a failure of the mechanisms that ensure immune tolerance to self-tissues. Central tolerance, which develops in the thymus for T cells and in the bone marrow for B cells, involves the negative selection and elimination of cells with high affinity for autoantigens. In T1D, this process is disrupted due to insufficient expression of tissue-specific antigens (primarily insulin) in the thymus, allowing autoreactive clones to escape deletion and exit the primary lymphoid organs [57]. Furthermore, polymorphisms in the *PTPN22*, *CTLA4*, and *IL2RA* genes affect the T-cell receptor activation threshold, weakening the efficiency of negative selection in the thymus and increasing the release of potentially pathogenic autoreactive cells into the peripheral immune system [48]. Mechanisms of peripheral tolerance, designed to control lymphocytes that have escaped central tolerance within secondary lymphoid organs and target tissues, include functional anergy, apoptosis, and suppression by Tregs. In T1D, a quantitative and/or functional deficit of Tregs is observed, along with increased resistance of effector T cells to their suppressive action, which disrupts the balance between immune response activation and inhibition [58]. As a result, lymphocytes specific to islet cell antigens retain their proliferative activity and pathogenic potential in the peripheral compartment.

The key link connecting autoreactive clones that have escaped immune tolerance to the initiation of tissue-specific inflammation is DCs. These professional antigen-presenting cells capture, process, and present β-cell autoantigens (insulin, GAD65, IA-2, ZnT8) in complex with MHC-II molecules to naive CD4^+^ T lymphocytes [59,60]. In the presence of genetic predisposition and an environmental trigger, DCs acquire a hyperactivated phenotype: they upregulate the expression of costimulatory molecules (CD80, CD86, CD40) and secrete pro-inflammatory cytokines: interleukin-12 (IL-12), IL-6, interferon-α (IFN-α), which skews T-helper (Th) differentiation toward T-helper 1 (Th1) and T-helper 17 (Th17) lineages, thereby promoting effector T-cell cytotoxicity [61]. The presentation of insulin and GAD65 by DCs in the pancreatic lymph nodes plays a decisive role in initiating the clonal expansion of autoreactive T cells. These cells subsequently migrate to the pancreas and form an inflammatory infiltrate in the islets of Langerhans, leading to progressive β-cell destruction [59]. Thus, the disruption of tolerance during lymphocyte selection, followed by the hyperactivation of DCs, creates the immunological foundation for the irreversible autoimmune destruction of pancreatic β-cells.

A key mechanism in the immunopathogenesis of T1D is the breakdown of central tolerance through the formation of neoantigens—modified or hybrid β-cell proteins that are absent from the thymus during T-cell selection. These include hybrid insulin peptides, generated by fusion of insulin fragments with other islet proteins within the processing compartments of antigen-presenting cells [62,63]. Additionally, under conditions of oxidative stress and local inflammation, native autoantigens undergo post-translational modifications, such as oxidation and deamidation, which radically alter their immunogenicity and enable their recognition by effector T cells [64]. One example is the C19S insulin B-peptide, recognized in the context of HLA-DQ8 by T cells [65]. Such peptides result from oxidative stress-induced modification of the cysteine thiol group. This process can occur during the disposal of unused secretory granules, which fuse with lysosomes to form crinosomes [66].

Viral burden also induces the expression of cryptic protein fragments and atypical structural variants of proinsulin, which serve as additional targets of the autoimmune attack [51,67]. It is precisely the presentation of these altered antigenic forms by DCs in pancreatic lymph nodes that drives clonal expansion of neoepitope-specific T cells and establishes a sustained pool of effector cells that migrate to the pancreas to execute inflammatory infiltration [68].

### 3.3. Effector Mechanisms of β-Cells Destruction

The direct destruction of insulin-producing cells is driven by the coordinated interaction of the cellular and humoral arms of the immune system, which together mediate the irreversible autoimmune destruction of pancreatic islet β-cells.

The T cell compartment serves as the primary effector arm of the autoimmune cascade. CD8^+^ cytotoxic T lymphocytes directly destroy β-cells through two main pathways: the release of perforin and granzymes, which form pores in the cell membrane—with limited damage triggering an apoptotic cascade via granzyme B-mediated activation of intracellular caspases, whereas extensive perforation leads to uncontrolled necrosis. In addition, FasL/Fas interaction between the lymphocyte and the β-cell induces an apoptotic signal through the extrinsic caspase 8 activation pathway [4,69].

CD4^+^ Th1 have traditionally been viewed not as direct effectors of destruction but rather as orchestrators of the immune response. By releasing IFN-γ, they activate macrophages and enhance antigen presentation on β-cells via MHC-I. Furthermore, IFN-γ drives ectopic expression of MHC-II on β-cells, converting them into non-professional antigen-presenting cells, which in turn may stimulate autoantigen-specific CD4^+^ Th [70,71]. Collectively, these processes create a sustained proinflammatory milieu that renders the islets highly vulnerable to CD8^+^-mediated destruction [4].

It should be noted, however, that direct cytotoxicity of CD4^+^ T cells via perforin granzyme and Fas/FasL pathways has been demonstrated in other autoimmune conditions, most notably multiple sclerosis, where CD4^+^ cells are capable of directly damaging oligodendrocytes [72,73]. In the context of T1D, however, a direct cytotoxic role for CD4^+^ cells remains unproven, and their contribution to β cell destruction is considered to occur primarily through regulatory and proinflammatory mechanisms.

The immunopathological cascade is further amplified by Th17 cells, which produce interleukin-17 (IL-17) and thereby stimulate pancreatic epithelial and stromal cells to secrete chemokines (CXCL1, CXCL8) that actively recruit neutrophils to the inflammatory site. Infiltrating neutrophils release reactive oxygen species and proteolytic enzymes, exacerbating oxidative stress and accelerating islet tissue destruction [74].

The B-cell arm in T1D primarily serves regulatory and marker functions [4]. B lymphocytes produce autoantibodies against insulin, GAD65, IA-2, and ZnT8, which serve as highly specific diagnostic markers of the autoimmune process and predictors of the disease progression rate [75]. However, research data clearly indicate that the autoantibodies themselves are not the primary effectors of tissue damage: they do not directly trigger β-cell destruction, but rather reflect the activation of the B-cell compartment. This compartment sustains chronic inflammation through the release of cytokines and participates in the presentation of islet autoantigens to CD4^+^ Th cells, forming a cycle of mutual B- and T-lymphocyte activation [76].

The culmination of this pathogenic cascade involves activated M1-polarized macrophages that infiltrate the islets of Langerhans and secrete high concentrations of pro-inflammatory cytokines, including interleukin-1β (IL-1β), tumor necrosis factor alpha (TNF-α), and IL-6 [77]. These mediators induce the expression of iNOS in β-cells, leading to the accumulation of toxic nitric oxide and the disruption of mitochondrial function [78]. IL-1β and TNF-α directly activate caspases and suppress the expression of genes responsible for insulin secretion, triggering apoptosis even in cells that have not yet been subjected to direct T-lymphocyte attack. IL-6, in turn, sustains chronic inflammation, stimulates the proliferation of effector T cells, and suppresses the generation of regulatory cell populations, thereby closing the loop of uncontrolled destruction [78,79].

The pathological process in the pancreas occurs in stages: from initial perivascular infiltration by immune cells to the subsequent penetration of lymphocytes and macrophages into the islet parenchyma, culminating in irreversible destruction and the complete loss of endocrine function [77]. Given the gradual nature of this process, the concept of preserved β-cell function defines early therapeutic intervention as a critical “window of opportunity”: maintaining even minimal endogenous insulin secretion is associated with better glycemic control, a reduced risk of severe hypoglycemia, and a delay in the development of vascular complications [80,81]. To evaluate the dynamics of autoimmune aggression, validated biomarkers are used: islet autoantibody titers (GAD65, IA-2, ZnT8) reflect the activity of the humoral arm; C-peptide levels serve as the gold standard for measuring preserved β-cell mass; and the immunological profile (Treg/Th17 ratio, cytokine status) allows for the monitoring of responses to immunomodulatory therapy [75,82]. Understanding these stages and biomarkers forms the pathophysiological basis for the use of mesenchymal stromal cells, whose mechanisms of action in preclinical and clinical studies are directed precisely at interrupting this cascade during the initial stages of β-cell destruction and ensuring the long-term preservation of pancreatic islet function.

## 4. Regenerative and Immunomodulatory Properties of MSCs

MSCs were first isolated from the bone marrow [83], and consequently, bone marrow-derived MSCs (BM-MSCs) are currently the most thoroughly characterized, making them a “gold standard” for describing the properties and functions of this cell type. MSCs are defined as cells meeting the following criteria: the ability to adhere to plastic after isolation, the capacity for multilineage differentiation into mesodermal lineage cells (adipocytes, chondrocytes, osteocytes) in vitro, surface expression of CD90, CD73, and CD105 markers, and the absence of hematopoietic marker expression (CD11b, CD14, CD34, CD45, HLA-DR) [84]. Cells with these characteristics can be isolated from virtually all mammalian organs and tissues, including adipose tissue, lungs, liver, extraembryonic tissues (umbilical cord, placenta, amniotic fluid), synovial fluid, dental pulp of deciduous teeth, and others.

In addition to the previously described characteristics, MSCs possess a range of other properties that make them a promising therapeutic tool for treating various diseases. In particular, MSCs exhibit significant regenerative potential. By migrating to the site of injury, they can initiate processes aimed at tissue repair, primarily by suppressing the death of resident cells, stimulating their proliferation and the differentiation of resident stem and progenitor cells, inducing angiogenesis, and exerting a protective effect on the damaged tissue cells [85,86]. The most therapeutically important feature of MSCs is their pronounced immunomodulatory capacity [87,88]. Numerous studies have demonstrated their ability to suppress the proliferation and activation of T lymphocytes and stimulate the expansion of Tregs [89]. MSCs suppress the proliferation of NK cells, induce their apoptosis, modulate cytokine secretion (IFN-γ and TNF-α), enhance degranulation, and inhibit cytotoxic function [90]. MSCs stimulate the metabolic reprogramming of macrophages, resulting in a phenotypic shift from the pro-inflammatory M1 state to the anti-inflammatory/pro-regenerative M2 phenotype [91,92]. They also exert a wide range of other effects on immune cells, ultimately leading to the fine-tuning of immune-mediated processes including T1D (Table 1).

Accumulating evidence suggests that these MSC effects, whether regenerative or immunomodulatory, are mediated largely through paracrine mechanisms, i.e., via the MSC secretome. The MSC secretome comprises a diverse array of cytokines, chemokines, growth factors, extracellular matrix proteins, and extracellular vesicles (EVs) including exosomes, microvesicles, and apoptotic bodies [93]. The therapeutic efficacy of the MSC secretome, including conditioned media and isolated EVs, has been shown to be comparable to that of the parent cells [94,95]. Depending on culture conditions or specific preconditioning, the MSC secretome can be directed to achieve more pronounced therapeutic effects [96,97]. Methods for MSC preconditioning have been developed to enhance the secretion of EVs with specific compositions and properties [98].

Although MSCs isolated from different tissues generally share common properties, tissue-specific characteristics related to their origin cannot be excluded. Indeed, depending on their tissue source, MSCs may differ in surface marker expression levels, proliferation rates, and the ability to differentiate into mesodermal lineage cells. For example, BM-MSCs efficiently differentiate in all three directions: osteogenic, adipogenic, and chondrogenic. In contrast, umbilical cord MSCs (UC-MSCs) have very low osteogenic potential, while liver-derived MSCs differentiate efficiently in the osteogenic and chondrogenic directions but show almost no adipogenic differentiation. The spectrum of secreted factors and the immunomodulatory properties can also vary significantly among different MSC types [99,100,101,102]. Using RNA sequencing methods, including bulk RNA-seq, scRNA-seq, and snRNA-seq, it has been shown that MSCs isolated from different tissues exhibit specific gene expression profiles with a substantial set of differentially expressed genes. This is likely related not only to their lineage commitment based on tissue origin but also defines their narrower functional specialization, which can significantly influence their therapeutic potential in various pathologies [103,104,105].

As immunomodulators, MSCs have been widely utilized in the development of both cell-based and cell-free products (such as conditioned media and EVs) for the treatment of various chronic inflammatory and autoimmune diseases [106]. In particular, promising preclinical and clinical results have been achieved in the treatment of rheumatoid arthritis [107,108], multiple sclerosis [109,110], type 1 diabetes [111,112,113], colitis [114,115], and other conditions. In the context of research on T1D therapy using MSCs and their secretome (exosomes, conditioned media), three main research directions can be identified: (1) MSCs as “chaperones” that support the engraftment of transplanted β-cells; (2) MSCs as stimulators of regeneration that exert a protective effect on β-cells; and (3) MSCs as immunomodulators that negatively regulate autoimmune processes.

Islet cell transplantation in T1D is an effective treatment strategy, and its outcomes continue to improve annually. However, the isolation of human islets significantly impairs their functional activity. Moreover, following transplantation, they are exposed to a hostile microenvironment characterized by inflammation, hypoxia, and elevated glucose levels, which substantially reduces their engraftment and survival [116,117]. Numerous studies have demonstrated that the co-transplantation of islets with MSCs significantly improves their engraftment and preserves functional activity, owing to the protective and immunomodulatory effects of the MSCs [118,119,120]. Furthermore, preconditioning islet cells with a cocktail of biologically active molecules secreted by MSCs has been shown to protect β-cells from inflammatory cytokine-induced apoptosis and enhance insulin secretion both in vitro and in vivo [121]. MSC-derived EVs have also exerted a beneficial effect on the function and survival of pancreatic islet cells. They protect against apoptosis and enhance insulin secretion by delivering biologically active components to recipient cells, including C3, ANXA1, SDF-1/CXCL12, and microRNAs (such as miR-21a-5p) [122]. Thus, the regenerative and immunomodulatory properties of MSCs—including their ability to protect islet cells from apoptosis, stimulate angiogenesis, and suppress autoimmune aggression—provide a compelling theoretical rationale for the application of MSC-based therapy in T1D.

However, following transplantation, MSCs are no longer in a controlled culture environment, but rather in the hostile internal milieu of the body, which is characterized by chronic hyperglycemia, oxidative stress, and systemic inflammation. Understanding how the diabetic microenvironment affects the viability, functional activity, and therapeutic potential of transplanted MSCs is a critical prerequisite for a realistic evaluation of the prospects for this approach.

## 5. Transplanted MSCs in the Diabetic Microenvironment

After grafting, transplanted MSCs enter an environment markedly different from cell culture conditions. Moreover, the parameters of this environment depend on the patient’s clinical status. In patients with diabetes, elevated glucose concentrations in body fluids and tissues represent the main hallmark of the internal milieu, which may affect the viability of transplanted cells and the overall efficacy of cell-based therapy. In healthy individuals, glucose levels are maintained within a narrow physiological range to ensure optimal uptake and utilization by all cells. For healthy young adults, the World Health Organization defines the reference range for fasting venous serum glucose as 3.9–5.5 mM [123]. For adults aged over 60 years, the recommended limit is 6.1 mM. Immediately after a meal, glucose concentrations rise and may double within 2 h, after which they gradually return to baseline levels, occasionally reaching 12–13 mM. In the cerebrospinal fluid of healthy adults, glucose concentration is 2.8–3.9 mM—lower than in plasma—due to the glucose concentration gradient between blood and brain parenchyma maintained by the blood–brain barrier [124]. Similar barriers regulating glucose uptake are also present in other tissues [125]. In untreated patients with diabetes, fasting serum glucose levels typically exceed 10 mM and may rise above 25 mM, but can be lowered with appropriate therapy. For patients with diabetes on glucose-lowering therapy, the current target range for fasting serum glucose is 4.6–7.1 mM [123].

MSCs, like other cells, depend on a supply of nutrients, including glucose, which is essential for energy production. MSCs can be maintained in vitro in culture media with widely varying glucose concentrations. For example, DMEM, the most widely used commercially available MSC culture medium, was initially formulated with a glucose concentration of 5.6 mM, mimicking physiological plasma levels. Later, a high-glucose formulation of DMEM containing 25 mM glucose was introduced. Culturing various cell types in high-glucose DMEM and other high-glucose media has been proposed as an in vitro model of diabetes [126]. It has been argued that, because of their highly glycolytic phenotype, MSCs require large amounts of glucose for normal growth in culture, are strongly dependent on glucose supply, and are sensitive to glucose depletion in the medium [127]. However, this view overlooks that high-glucose concentrations in the culture medium affect MSCs in multiple ways, many of which appear to be unfavorable. The overall effect depends on the MSC tissue source, oxygen supply, concentrations of other nutrients, and the overall experimental design.

Although an early study reported no immediate effects of high glucose on the proliferation or growth factor production of cultured human BM-MSCs [128], it later became clear that low-glucose medium enhances colony-forming capacity and reduce apoptosis in primary human BM-MSC cultures, whereas high glucose exerts the opposite effects and promotes cellular senescence [129,130]. In addition, high glucose was reported to impair the differentiation potential of primary MSCs. Interestingly, both of these studies demonstrated that high glucose enhanced the proliferation of MSCs immortalized by telomerase reverse transcriptase (TERT). Later, Chang et al. [131] confirmed these results in primary MSCs and also demonstrated that high glucose activates autophagy. Both short- and long-term exposure of human BM-MSCs to high glucose inhibited proliferation and limited their adipogenic differentiation potential [132]. In contrast, endothelial progenitor cells were resistant to acute or chronic high-glucose challenge.

Another study reported inhibition of proliferation, enhancement of adipogenic differentiation, and upregulation of several adipogenesis-related genes in cultures of MSCs derived from human bone marrow and several gestational tissues—chorion, placenta, and umbilical cord [133]. Osteogenic differentiation remained unchanged, although several related genes were downregulated. Culturing of human nucleus pulposus-derived MSCs in high-glucose medium resulted in decreased proliferation, colony formation, migration, and wound-healing activity, along with increased apoptosis, senescence, and caspase-3 expression, as well as suppression of stemness-related genes and downregulation of SIRT1, SIRT6, HIF-1α, and GLUT-1 at both the mRNA and protein levels [134]. Osteogenic differentiation of MSCs isolated from the buccal fat pad, as assessed by osteogenic marker expression, calcium deposition, and alkaline phosphatase activity, was significantly more pronounced in low-glucose (5.5 mM) than in high-glucose (25 mM) conditions [135]. The adverse effects of high glucose were attenuated by the addition of platelet-rich plasma. High glucose impairs mitochondrial function in human adipose-derived MSCs by disrupting mitochondrial membrane potential and the NAD^+^/NADH pool, and also interferes with mTOR-dependent regulatory pathways [136]. These studies were carried out under normoxic conditions. Since energy production in MSCs is largely glycolytic, the response of MSCs to high glucose may differ under hypoxic conditions. Notably, an earlier study claimed that high glucose supports BM-MSC survival and proliferation under severely hypoxic conditions [137]. However, the validity of this notion was never confirmed.

MSCs possess pronounced immunomodulatory activity, which is their most pivotal property from a translational perspective and the basis for many clinical applications currently in development. It has been shown that high glucose inhibits the immunomodulatory properties of human adipose-derived MSCs and makes them more immunogenic [138]. In this setting, the decline in MSC immunosuppressive capacity may be attributed to reduced levels of indoleamine 2,3-dioxygenase (IDO), interleukin-10 (IL-10), and complement factor H, along with increased immunoproteasome activity. High glucose downregulated levels of AMP-activated protein kinase (AMPK) and phosphofructokinase-1 (PFK-1), both of which regulate glycolysis. Taken together, these findings may explain the poor survival of transplanted MSCs and the occasionally disappointing clinical outcomes of MSC-based therapy in diabetic patients. This is consistent with reports showing toxic effects of hyperglycemia on human and rodent MSCs both in vitro and in vivo, possibly through modulation of the Wnt/β-catenin, PI3K/Akt/NF-κB, MAPK, and PKC pathways, as well as through alterations in microRNA expression [139,140,141].

In the clinical setting, MSCs can encounter a high-glucose environment in several scenarios. First, MSCs can have a long history of exposure to high glucose within the T1D patient’s body. There is evidence that MSCs isolated from patients with diabetes show a substantially reduced capacity to suppress immune cell activity, primarily due to low anti-inflammatory and high pro-inflammatory factor expression [142,143]. However, this scenario applies only to autologous transplantation, which is less common than allogeneic MSC therapy. Second, after infusion, transplanted MSCs are exposed to a persistently or intermittently hyperglycemic environment. In this case, the exposure period would be limited to the post-infusion lifespan of MSCs, which is rather short. Third, some MSC expansion protocols may include steps that use high-glucose media, potentially affecting the properties of MSCs intended for transplantation. As discussed above, a high-glucose environment both in vitro and in vivo may alter the basic properties of MSCs, potentially influencing the efficacy of cell therapy; however, the impact of MSC exposure to high glucose may vary across these three scenarios.

The post-infusion exposure of transplanted MSCs to a hyperglycemic environment may be the most significant of these scenarios. Some in vitro effects of high glucose on MSCs— such as impaired colony-forming capacity, reduced proliferation, altered lineage differentiation, cellular senescence, and persisting metabolic memory—take days to weeks to manifest. Given the short post-transplantation lifespan of MSCs, with peak cell death at 4 h [144], hyperglycemia is unlikely to alter the immunological properties of viable cells. The only real effect may be a modest stimulation of apoptosis, typical of high-glucose exposure [136]. Potentially more important is how high glucose affects the interactions of immune cells with dying MSCs and EVs, including apoptotic bodies, which may contribute to MSC-mediated immunomodulation [93,94,98,145]. High-glucose levels enhance glycolysis in macrophages, leading to lactate accumulation and increased CD11b lactylation, which directly inhibits efferocytosis [146]. In addition, hyperglycemia appears to reduce the expression of CD36, a key receptor for apoptotic cell recognition, through a SIRT6–miR-217 mechanism [147]. Efferocytosis by DCs is also substantially impaired in the diabetic environment, which is associated with increased SLC7A11 expression [148]. Moreover, CCR7-dependent DC migration to lymph nodes is reduced [149], which may limit peripheral tolerance induction by tolDCs. Finally, there is evidence that, under high-glucose conditions, MSCs may produce EVs with pro-inflammatory and damaging rather than immunosuppressive properties [150]. It is possible that all these immunological processes associated with high-glucose levels influence the efficacy of MSC-based therapy. If so, strict glycemic control in patients should be a prerequisite for successful treatment.

There is evidence that the use of high-glucose culture media during cell product manufacturing may affect the immunological properties of the cells. Indeed, adipose-derived MSCs cultured under high-glucose conditions (25 mM) show reduced immunomodulatory capacity, associated with markedly lower IDO and IL-10 levels. In addition, the observed substantial increase in immunoproteasome activity, which is involved in antigen processing and MHC-I presentation, may render MSCs more immunogenic [138]. Moreover, high-glucose levels are likely to affect the properties of the cells by modifying long-lived macromolecules through glycation and glyoxidation, which can lead to loss of their function and oxidative stress [151,152,153]. The effects of high glucose on MSC properties should be taken into account when developing protocols for clinical-grade cell production, since some media used in biomanufacturing (DMEM (Hi), GMEM, IMDM) contain elevated glucose levels. Notably, the effects of high glucose on MSCs may be at least partly reversible. Tan et al. [154] induced accelerated aging in human synovial MSC cultures by exposing them to high-glucose medium (25 mM) for 24 h and then returning them to normal conditions (5 mM) for an additional 24 h. High-glucose-induced cellular senescence, as evidenced by upregulation of senescence-associated secretory phenotype (SASP) genes, an increased number of senescence-associated β-galactosidase (SA-β-gal)-positive cells, elevated mitochondrial ROS levels, and enhanced mitochondrial fission was largely reversed by subsequent incubation in low-glucose medium for 24 h.

## 6. Preclinical Assessment of MSC-Based Immunotherapy in T1D Animal Models

The most relevant animal model of T1D is the non-obese diabetic (NOD) mouse, in which multiple mutations drive an autoimmune response and insulitis, leading to spontaneous disease development [155]. Alternatively, diabetes can be induced by intraperitoneal administration of streptozotocin (STZ) [156]. A single high-dose administration of STZ rapidly destroys β-cells, causing hyperglycemia. This approach is typically used to model late diabetic complications or for islet cell transplantation studies. In contrast, multiple low-dose administrations of STZ (MLD-STZ), e.g., 30–50 mg/kg daily for 5 days, trigger a T-cell-mediated autoimmune response, leading to insulitis and slow destruction of β-cells. Although the MLD-STZ model is not fully equivalent to the spontaneous NOD mouse model because it relies on a chemical trigger, it is widely used for T1D modeling given its lower cost and reduced time requirements.

In various animal models, MSC transplantation has been shown to protect against islet deterioration by reducing β-cell death, normalizing glycemic levels, enhancing β-cell proliferation, and stimulating endogenous regeneration [157]. Gao et al. [158] demonstrated that MSCs engineered to overexpress SPARC (secreted protein acidic and rich in cysteine), when administered intravenously to mice with MLD-STZ-induced diabetes, significantly reduced hyperglycemia, ameliorated pancreatic islet damage, and promoted the recovery of β-cell function and insulin production. SPARC regulates β-cell survival, and its overexpression in β-cells cultured under high-glucose conditions enhances insulin secretion [159,160]. Exosomes derived from MSCs and loaded with nanoselenium also exerted a protective effect in the MLD-STZ-induced T1D rat model. They substantially reduced oxidative stress in the islets, increased intracellular antioxidant levels, markedly suppressed islet cell apoptosis, and restored islet architecture [161].

Some studies suggest that MSC-derived exosomes can slow down or even halt the progression of MLD-STZ-induced diabetes in rat and mouse models by inhibiting the autoimmune destruction of pancreatic islets, promoting the apoptosis of autoreactive T cells and the proliferation of Tregs. These effects lead to enhanced islet regeneration, angiogenesis, and survival, ultimately increasing insulin secretion and controlling hyperglycemia and its complications [162,163]. Furthermore, MSC-derived exosomes have been shown to functionally restore existing islets [160,164]. Beyond their effects on islets and β-cell function, MSC transplantation also promotes at least partial recovery of other organs damaged during T1D progression, including the liver, kidneys, and spleen [165].

Many studies on MSCs in T1D animal models have focused on their immunomodulatory role. Preclinical data suggest that MSCs can slow down or even reverse the progression of diabetes in NOD mice [166,167]. In addition to suppressing pathogenic CD8^+^ T cells, Th1, and Th17 [168,169], MSCs can also promote the expansion of Tregs through paracrine effects, direct interaction with cellular receptors, or mitochondrial transfer [170]. For instance, Zhang et al. [171], comparing the therapeutic efficacy of BM-MSCs and UC-MSCs administered intravenously to NOD mice with MLD-STZ-stimulated diabetes, showed that the main mechanism underlying MSC-mediated remission is primarily related to the modulation of autoimmune islet destruction. Both cell types significantly attenuated insulitis in the islets, reduced the number of Th17 (IL-17^+^ CD4^+^), and increased the number of Tregs (FOXP3^+^ CD4^+^) in the spleen and lymph nodes [171]. An increase in the number of Tregs was also observed in rats with MLD-STZ-induced diabetes following the intraperitoneal injection of conditioned media obtained from 2D or 3D UC-MSC cultures [172]. Wu et al. [173] found that *TGFBI* gene is essential for the immunomodulatory function of UC-MSCs in MLD-STZ-induced mouse model. Following intravenous administration, UC-MSCs with low *TGFBI* expression exhibited reduced immunosuppressive capacity and a weakened therapeutic effect, manifested by increased pathological islet damage, a higher number of infiltrating T cells in the pancreas, and increased expression of IFN-γ and IL-17A. Conversely, TGFBI produced by UC-MSCs suppressed the proliferation of activated T cells by inhibiting Cyclin D2 expression. TGFBI is an extracellular matrix protein that plays a vital role in ensuring the survival and function of the islets of Langerhans and in inhibiting T-cell activation [174,175]. The important role of transforming growth factor-β (TGF-β) in the immunomodulation mediated by transplanted MSCs in the MLD-STZ-induced diabetes mouse model was also demonstrated by Du et al. [176], who established that 3D culture of UC-MSCs triggers their transcriptomic reprogramming toward a YAP1 state activated by the Hippo signaling pathway, accompanied by enhanced release of TGF-β1 [174].

Lachaud et al. [177], using the RIP-B7.1 mouse model of experimental autoimmune diabetes induced by injection of a preproinsulin (ppINS) expression plasmid—a model that accurately mimics the aggressive immune attack observed in young children with T1D [178]—showed that intraperitoneal injection of UC-MSCs led to a pronounced recruitment of myeloid-derived suppressor cells (MDSCs) into the peritoneal cavity. This was followed by multiple immunosuppressive responses of T, B, and myeloid cells in the peritoneal fluid, spleen, pancreatic lymph nodes, and pancreas, resulting in a significant reduction in insulitis and pancreatic infiltration by T and B cells, as well as pro-inflammatory macrophages. A single dose of UC-MSCs provided the best protection against the development of insulitis, preserved islet integrity and normal α- and β-cell content, reduced plasma concentrations of pro-inflammatory cytokines IL-6, IFN-γ, and TNF-α, and significantly reduced the infiltration of CD4^+^ and CD8^+^ T cells, CD19^+^ B cells, and CD11b^+^ myeloid cells into the pancreas. A notable finding is that UC-MSCs transplanted into RIP-B7.1 mice were extensively exposed to CD11b^high^ peritoneal macrophages, leading to the formation of peritoneal UC-MSC/macrophage aggregates, which were eliminated from the abdominal cavity via phagocytosis [174]. Despite their rapid clearance from the body, the transplanted UC-MSCs significantly altered the cellular composition and inflammatory state of resident immune cells in the peritoneal fluid, triggering a rapid and massive mobilization of MDSCs into the peritoneal cavity, known to play a critical role in the resolution of acute inflammation [179]. Furthermore, UC-MSC transplantation caused a significant increase in the expression of arginase-1 (ARG1) in the peritoneal fluid. ARG1 is an enzyme with potent immunosuppressive activity, highly expressed by mouse MDSCs and anti-inflammatory M2 macrophages during the resolution phase of inflammation [180,181]. As observed by the authors, the next stage in the anti-inflammatory response to UC-MSC administration was the intraperitoneal mobilization of Tregs and their expansion in the pancreatic lymph nodes and spleen. Thus, UC-MSCs induced several waves of immune response, shifting the aggressive autoreactive profile of immune cells toward an anti-inflammatory and regenerative state.

In addition to umbilical cord and adipose MSCs, which are the most frequently investigated in the context of T1D therapy [182], MSCs derived from other tissue sources also seem to exhibit pronounced immunomodulatory effects in animal models. These include MSCs from the outer root sheath of the hair follicle [183], menstrual blood-derived endometrial MSCs [165], and others.

To enhance the immunomodulatory properties of MSCs in the context of diabetes therapy and other immune-mediated diseases, preconditioning is often applied. For example, preconditioning MSCs with hypoxia, IFN-γ, poly(I:C), or LPS significantly increases the immunomodulatory activity of both the cells themselves and their derived EVs in the MLD-STZ-induced T1D mouse model [184,185]. Preconditioning or functionalization of MSCs with nanoparticles containing silymarin, silybin, and selenium—substances with anti-inflammatory and antioxidant properties—mitigated inflammatory processes and oxidative stress in BM-MSCs, thereby enhancing their immunomodulatory properties through the inhibition of the PI3K/AKT/NF-κB signaling pathway [186]. 3D culturing of MSCs as aggregates on low-adhesion surfaces enhances their immunomodulatory properties and greatly increases EV production [187,188]. Ren et al. [189] demonstrated that EVs derived from aggregated MSCs inherit lactate from the parent cells and, upon being taken up by CD8^+^ T cells, enhance L-cysteine breakdown within these T cells. Consequently, L-cysteine metabolism is upregulated in CD8^+^ T cells, leading to decreased levels of L-cysteine, glutathione (GSH), and the GSH/GSSG (glutathione disulfide) ratio, which in turn results in increased hydrogen sulfide (H_2_S) levels. H_2_S can activate β-catenin, increasing the expression of programmed cell death protein 1 (PD-1) and thereby suppressing the proliferation and function of CD8^+^ T cells. When administered to animals with T1D, such EVs led to the depletion of the CD8^+^ T-cell pool and improved β-cell function [187].

Overall, preclinical studies of MSC-based T1D therapy indicate that the field has made substantial progress over the past decade. Many of the mechanisms behind the therapeutic effects of MSCs are now understood, and ways to enhance these effects have been developed. Ultimately, these findings may provide a foundation for developing more effective cell-based or cell-free products for treating T1D.

## 7. Clinical Studies of Immunomodulatory Therapy with MSCs in T1D

Extensive preclinical studies in animal models of T1D have demonstrated that MSCs not only support β-cell survival but also actively modulate the pathogenic cascade: suppressing islet infiltration, mitigating local inflammation, and promoting peripheral tolerance. These findings have laid the foundation for translating MSC-based therapies into clinical practice. Clinical trials of MSC-based immunomodulatory therapy in T1D began more than a decade ago and are ongoing. This trend reflects a shift from attempts to induce pancreatic β-cell regeneration toward immunomodulatory strategies aimed at suppressing the autoimmune attack and preserving residual β-cell function.

An analysis of protocols registered on ClinicalTrials.gov [190] and published research articles reveals significant design heterogeneity (Table 2). The studies employ MSCs from various autologous and allogeneic tissues: bone marrow, adipose tissue, umbilical cord, including Wharton’s jelly. Administration strategies range from single infusions to multiple courses as well as dose-escalation designs. Most protocols use systemic intravenous administration. There are attempts to employ targeted, superselective intra-arterial infusion directly into the pancreatic artery to maximize the localization of the cellular product in the zone of active infiltration and destruction of the islets of Langerhans [191,192].

In most of the analyzed protocols, the immunomodulatory effect is declared as the primary hypothesis or included in the key endpoints. It is hypothesized that MSCs secrete a wide range of paracrine factors: prostaglandin E2 (PGE2), IDO, TGF-β, IL-10, exosomes with regulatory miRNAs that induce macrophage polarization from the pro-inflammatory M1 phenotype to the anti-inflammatory M2 phenotype, suppress the proliferation of Th1/Th17, and promote the expansion of CD4^+^FoxP3^+^ Tregs. Even in the absence of direct β-cell regeneration, the creation of a local immunosuppressive microenvironment in the islets of Langerhans and pancreatic lymph nodes allows for the interruption of the pathogenic cascade of autoimmune aggression and the preservation of residual β-cell function.

The analyzed parameters demonstrate clear stratification. Standard metabolic and clinical markers are universally used across all studies: C-peptide levels (basal and stimulated after MMTT/OGTT), glycated hemoglobin (HbA1c), daily exogenous insulin requirement, frequency of hypoglycemic events, and safety profile (recording of adverse events). Less frequently, but with a clear focus on pathophysiology, protocols include specific immunological and technologically complex indicators: islet autoantibody titers (GAD65, IA-2, ZnT8), lymphocyte subpopulation phenotyping (Treg/Th17 ratio, direct measurement of CD4^+^FoxP3^+^), cytokine profiles, and continuous glucose monitoring (CGM) data with calculated glycemic variability indices. Some studies extend beyond short-term follow-ups, evaluating long-term microvascular complications over 5–8 years, which provides a new perspective for assessing the durability of the therapeutic effect.

Although most studies are in the recruitment or protocol planning stages, some published reports offer preliminary clinical evidence for the therapeutic potential of MSCs in T1D. A general trend is a significant reduction in the need for exogenous insulin and improved glycemic control within the first few months after administration, which is accompanied by a prolongation of the partial remission phase (“honeymoon period”) in the vast majority of patients in the therapy groups. By the six-month follow-up, a significant increase in basal C-peptide is recorded, indicating the preservation of residual β-cell function. In patients with severe onset, including diabetic ketoacidosis, UC-MSCs and BM-MSCs have demonstrated comparable efficacy. This is supported by both clinical and parallel preclinical data showing identical reductions in insulitis, a decrease in the Th17 lymphocyte pool, and an increase in Tregs. Randomized trials confirm that stimulated C-peptide is maintained or increased in the therapy groups, whereas the expected progressive loss of β-cell function occurs in the control groups; meanwhile, no serious therapy-related adverse events have been reported. The most informative long-term data, with follow-up of up to eight years, show that combination therapy with targeted intra-arterial delivery provided not only sustained improvement in metabolic parameters in the first year but also a statistically significant reduction in the incidence of microvascular complications—peripheral neuropathy and diabetic nephropathy—compared to the control group. This indicates a prolonged immunometabolic effect that extends beyond short-term metabolic correction.

Of particular interest are the data from several studies indicating that the effects of MSC therapy in T1D may persist for prolonged periods. Clinical protocols generally do not involve extended treatment courses and are typically limited to one or several cell injections (Table 2). Nevertheless, therapeutic effects persisted throughout the entire follow-up period, in some cases for up to 7 years [191,192,193,194]. However, allogeneic MSCs are short-lived in the recipient’s body, as shown in both animal models [195] and clinical studies [144]. Following intravenous administration, allogeneic MSCs accumulate in the lungs, with peak cell death at 4 h. Only a very small number of these cells then redistribute to the liver, spleen, kidneys, and other abdominal organs, where they persist briefly [196]. In this context, it seems implausible that allogeneic MSCs would home to the inflamed pancreatic tissue and sustain long-term immunosuppression via paracrine signaling. This apparent discrepancy is difficult to resolve without assuming the involvement of autologous long-lived immunological memory cells. It is possible that the massive death of transplanted MSCs and the resulting cell fragments, such as apoptotic bodies, may serve as a trigger for restoring immunological tolerance. Because pulmonary intravascular macrophages (PIMs) are scarce or entirely absent in human lungs [197], most apoptotic bodies may relocate to the spleen and liver, where they are efferocytosed by DCs and macrophages that acquire an immunosuppressive phenotype as a result of the suppressive microenvironment shaped by the apoptotic bodies [198]. Subsequently, tissue-resident tolDCs may migrate to the pancreatic draining lymph nodes and initiate peripheral tolerance through Treg promotion and the induction of anergy in effector lymphocytes. Notably, the ability of allogeneic MSCs to act through a “hit and run” mechanism [199] makes MSC-based therapy similar to immunization with a tolerogenic cell vaccine. If so, HLA differences between the donor and the recipient may affect not only MSC immunogenicity but also the broader immunological processes they trigger. In this regard, it would be of interest not only to compare the efficacy of allogeneic and autologous MSCs but also to assess their efficacy in the context of individual HLA allele matching. Of course, the suggested mechanisms are highly speculative, and large-scale clinical trials may fail to confirm the currently available fragmentary evidence for long-term MSC therapy effects.

An important question is whether there is a causal relationship between the delivery route and the possible clinical benefit. Intravenous administration remains the most common route of MSC delivery, likely owing to its simplicity, safety, versatility, and good reproducibility in standard therapeutic procedures. In contrast, intra-arterial delivery is a technically demanding procedure that is justified only for specific purposes and carries higher risks. At the same time, intra-arterial injection bypasses the pulmonary filter, enabling broader MSC distribution to other organs. A biodistribution study of ^18^F-FDG-labeled BM-MSCs in 21 patients with T1D demonstrated that intravenous delivery resulted in pulmonary trapping, with no uptake in the pancreas, whereas selective intra-arterial delivery led to MSC homing to the pancreatic head (via the pancreaticoduodenal artery) or the pancreatic body (via the splenic artery) [200]. The only clinical study using intra-arterial MSC delivery [191,192] showed encouraging results and, importantly, demonstrated a notable reduction in long-term complications of T1D (Table 2). However, it would be premature to draw conclusions about the benefits of intra-arterial MSC delivery for T1D treatment. A comparative clinical study with a standardized cell product and uniform testing conditions is required.

Accumulated clinical data indicate a favorable safety profile for MSC transplantation across various diseases. Despite several reports of thromboembolism and bacterial infection, systematic reviews and meta-analyses [201], including those focused on diabetes [202,203], have not revealed a statistically significant increase in the incidence of serious adverse events—including infections, thromboembolic complications, fatal outcomes, and malignancies—associated with MSC administration. Notably, however, that these data do not address long-term consequences, which remain to be evaluated. Late adverse events such as tumor transformation or incorporation of transplanted MSCs into tumor stroma are highly unlikely to occur because of the limited lifespan of administered MSCs. Remarkably, both allogeneic and autologous MSCs appear to have a limited post transplantation lifespan, as demonstrated in animal models [204,205]. Indirect clinical evidence also supports this assumption [206]. The main causes of transplanted MSC death are thought to be apoptosis triggered by the pulmonary microenvironment, detachment-induced cell death (anoikis), as well as metabolic and mechanical stress during vascular passage [207]. Immune clearance appears to play a secondary role in MSC survival, although activated NK cells are known to efficiently lyse both allogeneic and autologous MSCs [208]. Another concern is the possibility of allosensitization following allogeneic MSC infusion, which could complicate future transplantations, including islet grafts. This concern is based on data showing that allogeneic MSC administration in rats induces alloantibodies that mediate complement-dependent lysis, thereby shortening MSC survival upon subsequent infusions [209]. However, a systematic review including data from 356 patients who received allogeneic MSCs showed that only 11.51% of these patients developed donor-specific antibodies (DSAs). Moreover, DSA production did not correlate with safety or treatment tolerance, and neither the number of infusions nor the degree of HLA mismatch influenced antibody development [210]. Thus, potential alloantigen sensitization is more a matter of efficacy than of safety.

## 8. Therapeutic Potential of MSCs in Addressing Long-Term Complications of T1D

The risk of progressive damage to blood vessels, peripheral nerves, and kidneys (nephropathy, neuropathy, and retinopathy) remains substantially elevated in the long term, even when target metabolic goals are achieved. This is because insulin replacement therapy does not eliminate the chronic metabolic disturbances and systemic inflammation that underlie microvascular complications [211]. Exogenous insulin compensates for the hormone deficiency but does not suppress autoimmune activity or normalize the post-translational protein modifications that continue to drive endothelial dysfunction and axonal degeneration [212].

A key cross-reactive autoantigen linking pancreatic autoaggression to neurological damage is GAD65. In the pancreas, GAD65 is localized in the cytosol of β-cells, where it functions as an enzyme catalyzing the synthesis of γ-aminobutyric acid (GABA), and it serves as one of the primary targets of autoantibodies at the clinical onset of T1D [213]. Concurrently, GAD65 is expressed in GABAergic neurons of the central and peripheral nervous systems, where it acts as a key regulator of inhibitory processes and the maintenance of neurophysiological homeostasis [214]. This dual tissue localization is dictated by the evolutionarily conserved structure of the isoenzyme, rendering neurons vulnerable to cross-reactivity from antibodies initially formed against islet cells [215]. Current evidence indicates that the conformation-dependent GAD65 epitopes recognized in T1D are structurally homologous to those on neurons. Due to this similarity, antibodies generated against pancreatic GAD65 can bind directly to neuronal GAD65 without requiring additional affinity maturation [216]. Under conditions of systemic inflammation, high-affinity anti-GAD65 antibodies can cross the blood–brain barrier and bind directly to GABAergic neurons, disrupting synaptic transmission and contributing to the development of cognitive impairment and autonomic dysfunction [217]. As the disease progresses, the autoimmune response broadens to adjacent epitopes and related neural proteins, which markedly amplifies local neuroinflammation and accelerates axonal degeneration [218]. Recent data demonstrate that this process sequentially involves homologous neural proteins (including GAD67 and neurofilaments), which clinically correlates with the progression of neuropathy independent of current glycemic control [219]. Furthermore, a specific expansion of CD4^+^ T cells responding to GAD65 peptides with neuronal homology has been identified, definitively confirming molecular mimicry as a key mechanism of neuroimmune damage [220]. The accumulation of high-affinity antibodies promotes the formation of immune complexes, complement activation, and the recruitment of cytotoxic lymphocytes into the perineurium. Together, these processes drive the development of systemic polyneuropathy, significantly impairing patients’ quality of life and reducing their life expectancy [216].

Furthermore, the kidneys and retina are also targets of T1D-associated cross-reactive autoimmunity, where chronic inflammation and immune dysregulation play a role as significant as metabolic disturbances. Despite advances in glycemic control, diabetic nephropathy and retinopathy remain the leading causes of end-stage renal disease and irreversible vision loss, indicating the existence of glycemia-independent pathogenic mechanisms [221]. The pathogenesis of diabetic nephropathy has traditionally been linked to hemodynamic disturbances; however, current data emphasize the role of immune infiltration in the glomeruli and tubules. In the renal tissue of patients with T1D, increased expression of pro-inflammatory cytokines (IL-1β, TNF-α, IL-6, CCL2), along with infiltration by macrophages and T lymphocytes, is observed and correlates with the rate of decline in the glomerular filtration rate [222,223]. Although direct cross-reactivity of anti-GAD65 with renal antigens remains hypothetical, the overall autoimmune background creates favorable conditions for the progression of nephropathy, independent of the level of glycemic control [221].

Similar mechanisms are observed in the pathogenesis of diabetic retinopathy. In addition to classic factors (oxidative stress, protein kinase C activation, accumulation of advanced glycation end products), dysfunction of the blood–retinal barrier, mediated by the activation of resident microglia and infiltration by peripheral immune cells, plays a key role [224]. GAD65 is expressed in the amacrine and horizontal cells of the retina, where it participates in the GABAergic modulation of visual signals [225], theoretically rendering these structures vulnerable to cross-reactive antibody attacks. However, the direct pathogenic role of anti-GAD65 in the development of retinopathy remains unproven and requires further verification. It is well established that the systemic pro-inflammatory state in T1D (elevated levels of IL-6, TNF-α, MCP-1) promotes the progression of retinopathy independently of glycemic control [226]. Thus, persistent autoimmune aggression initiated in the pancreas forms a unified pathogenic cascade that affects the nervous system, kidneys, and retina through common immunological mechanisms. In this context, MSC-based therapy acquires a fundamentally new significance. Its immunomodulatory potential remains clinically relevant even in situations where the islet apparatus is completely destroyed, β-cells are absent, and the patient is entirely dependent on exogenous insulin [227].

**Figure 2:**
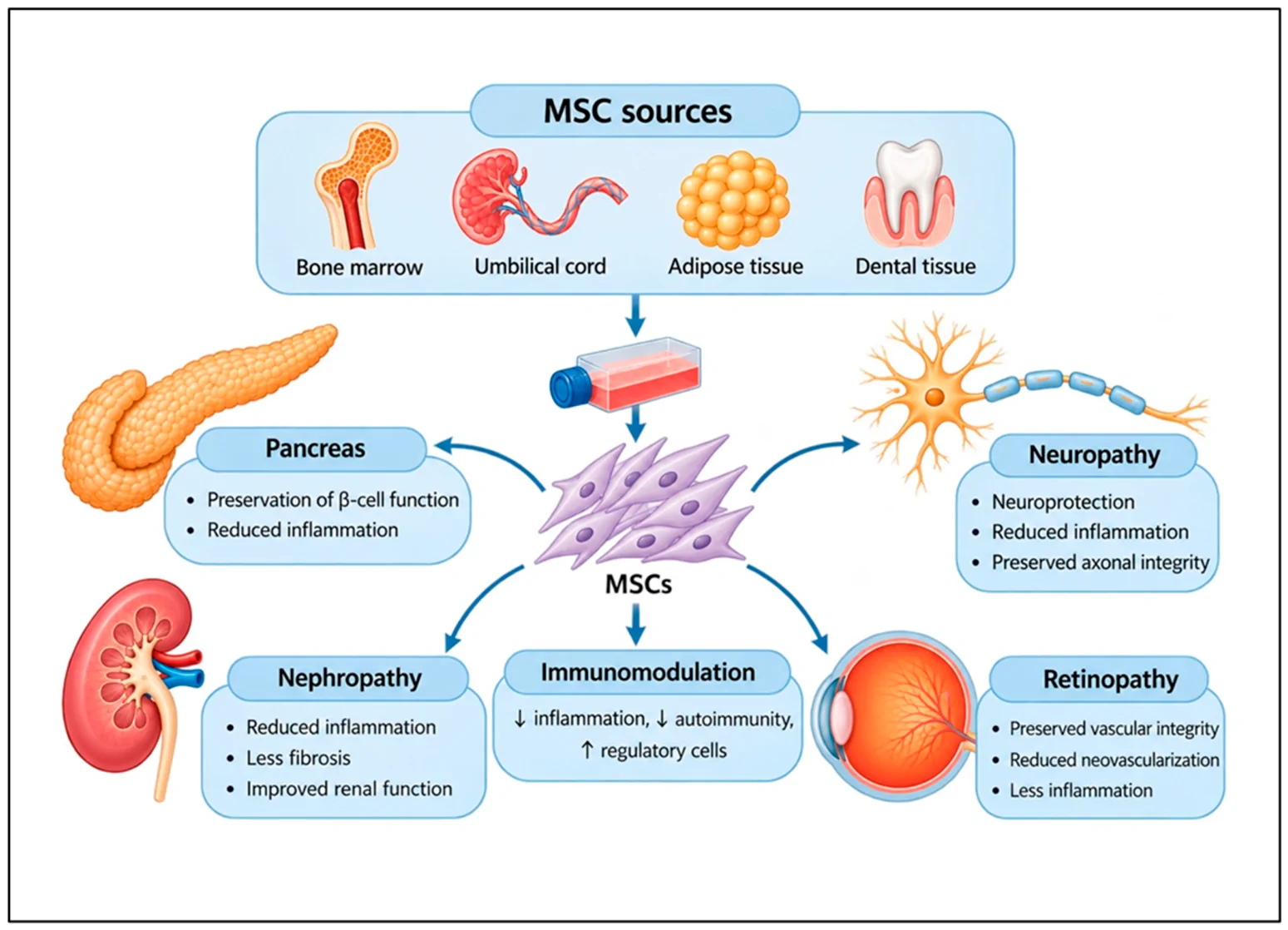
Mesenchymal Stromal Cells Target Multiple Pathological Processes in Type 1 Diabetes.

MSCs systemically suppress autoreactive clones, reduce titers of circulating anti-GAD65 antibodies, and block the neuroinflammation cascade through the paracrine secretion of IDO, PGE2, TGF-β, and exosomes containing regulatory microRNAs [228]. Recent preclinical data expand this paradigm, demonstrating that MSCs can exert local effects on the peripheral nervous system: they secrete neurotrophic factors (BDNF, NGF, GDNF), suppress the activation of resident macrophages in the endoneurium, and stimulate axonal remyelination, with these effects persisting even after the complete loss of β-cell mass [229] (Figure 2).

In the context of nephropathy, MSCs polarize macrophages from the pro-inflammatory M1 phenotype to the reparative M2 phenotype, reducing the production of fibrogenic cytokines (connective tissue growth factor (CTGF), TGF-β1) and slowing the development of glomerulosclerosis and tubulointerstitial fibrosis [230]. MSC-derived exosomes enriched with regulatory miRNAs (miR-146a, miR-21) suppress the activation of NF-κB and TGF-β/Smad pathways in mesangial cells, experimentally confirming the antifibrotic potential of cell therapy [231]. In retinopathy, MSCs exert a dual effect: they suppress pathological neovascularization by inhibiting VEGF signaling under hyperglycemic conditions, while simultaneously preserving endothelial integrity through the secretion of angiopoietin-1 and survival factors [232]. Furthermore, the established immunomodulatory properties of MSCs—reducing pro-inflammatory cytokine levels, polarizing macrophages toward the anti-inflammatory M2 phenotype, and expanding Tregs—create a favorable systemic background that may indirectly slow the progression of retinopathy [226,228]. However, a direct causal link between systemic MSC immunomodulation and a specific protective effect on kidney and retinal tissues in T1D remains speculative and requires further experimental and clinical verification. Critically, the described mechanisms do not require the trans-differentiation of MSCs into renal or neuronal cells. The therapeutic effect is realized through paracrine regulation of the microenvironment, making this therapy applicable even in the late stages of complications, when structural tissue restoration is biologically impossible. Interestingly, the only clinical trial that enrolled patients beyond the early stages of T1D, including those with a disease duration of up to 20 years (Table 2), yielded highly encouraging results for preventing long-term complications, including nephropathy and retinopathy [192].

## 9. Conclusions

MSCs represent one of the most promising platforms for cell therapy in T1D, capable of targeting key nodes of the autoimmune cascade—from antigen presentation and effector T-cell activation to sustained chronic inflammation in the islets of Langerhans. Although the number of completed clinical trials remains limited, they demonstrate consistent trends toward efficacy—prolongation of the partial remission phase, preservation of stimulated C-peptide levels, and reduction in exogenous insulin requirements. The most promising data, although preliminary, suggest that just a few MSC injections can yield lasting improvement. A decreased incidence of long-term microvascular complications in patients with long-standing T1D is also encouraging. These data suggest that MSC therapy may find its place among other modern biotherapeutic approaches targeting the immunopathological mechanisms driving T1D.

One of the earliest approaches in T1D immunotherapy involved monoclonal antibodies targeting the CD3 receptor (teplizumab). Anti-CD3 therapy modulates T-cell activity, pushing autoreactive clones into a state of functional unresponsiveness while promoting an expansion of Tregs that suppress autoimmune inflammation. Large-scale clinical trials have demonstrated that teplizumab can delay the progression of T1D by 2–3 years in individuals at high genetic risk [14]. Selective depletion of B lymphocytes with anti-CD20 antibodies (rituximab) is designed to interrupt their antigen-presenting function—through which B cells activate autoreactive Th. In a randomized phase II trial, rituximab showed statistically significant but transient preservation of C-peptide secretion. However, by 12 months, the beneficial effect gradually waned, as the therapy also depleted the pool of regulatory B cells, which are critically important for maintaining immune tolerance [15,233].

Apart from MSCs, “professional” immunosuppressive cells such as Tregs and tolDCs are being tested in clinical trials for T1D treatment. These trials have demonstrated favorable safety profiles and evidence of immunomodulatory activity, although their clinical efficacy remains uncertain. A randomized phase II trial involving 110 children and adolescents with new-onset T1D found that a single Treg infusion did not significantly preserve residual β-cell function over one year compared with placebo [234]. In a phase I trial involving nine adults with long-standing T1D, two administrations of proinsulin-loaded tolDCs induced a sustained reduction in proinsulin-specific autoreactive T-cell responses, detectable for up to three years after treatment [9]. However, convincing evidence of improved glycemic control or preservation of β-cell function is still lacking.

Clearly, both MSC-based therapy and other immunotherapeutic approaches to T1D are at early stages of clinical investigation, making direct comparison between them problematic. Larger controlled trials are therefore needed. However, it is already clear that MSCs offer important advantages, including their multimodal mechanism of action and the possibility of using allogeneic cells. The latter enables the development of standardized cell products and avoids the need to use the patient as a donor, whereas Treg- and tolDC-based approaches require patient-derived cells.

Over the past decades, the prevailing view of how MSCs exert their therapeutic effects has changed from replacing damaged host cells toward the view that MSCs act as a source of paracrine factors that stimulate resident stem cells and exert immunomodulation. This has driven the development of therapeutic approaches based on allogeneic MSCs, which continue to evolve today. Now, however, a second paradigm shift is emerging: a growing body of evidence suggests that the therapeutic activity of MSCs may not depend solely on living cells and that their fragments, such as extracellular vesicles and apoptotic bodies, can exert comparable effects. These findings provided a powerful stimulus for the development of cell-free approaches, in which MSCs serve as starting material for manufacturing MSC-derived cell-free products [235,236,237]. While the cell-free approach has substantial potential, directly comparing the clinical efficacy of MSC-derived cell-free products with that of living MSCs is challenging. Given that intravenous delivery is used in the vast majority of cases, we are, in effect, delegating cell-free product manufacturing to the recipient’s lungs, where most cells rapidly die, giving rise to cell fragments. In this context, particular attention should be given to testing autologous MSCs with intra-arterial delivery, a strategy that may extend MSC lifespan in the body.

A more detailed analysis of the MSC immunophenotype may be useful in clinical trials, especially in T1D. The HLA haplotype and the profile of immunosuppressive and immunostimulatory molecules of allogeneic MSCs should be taken into account. Importantly, these membrane molecules, including HLA, can persist on cell fragments and retain their immunological function [238,239]. It cannot be excluded that the degree of HLA matching influences the efficacy of cell-based therapy; moreover, this information would be useful for assessing allosensitization and the suitability of islet transplantation.

Despite the progress made in the field of MSC-based immunotherapy for T1D, several key questions remain unresolved: What are the optimal tissue source, cell dose, passage number, route of administration, and infusion frequency? How does the immunophenotype of MSCs influence their immunomodulatory properties? Which are more effective, allogeneic or autologous MSCs, given that autologous cells may be derived from patients with poorly controlled diabetes? Can one or a few administrations of MSCs produce long-term therapeutic effects, and if so, what are the underlying mechanisms? Do MSCs have the potential to prevent or delay diabetic complications in patients with advanced T1D, when no residual β-cell function remains? Addressing these and other questions will require further laboratory research and larger, well-designed clinical trials with clearly defined endpoints. Among the many outstanding questions, perhaps the most important is the following: Does the administration of live MSCs offer sufficient advantages over rapidly evolving cell-free approaches? The answer to this question will largely determine whether MSC-based cell therapy for T1D finds its way from bench to bedside.

## Figures and Tables

**Table 1:** Molecular mechanisms of MSC-mediated immunomodulation in T1D.

Targets of MSCs	Molecular Mediators	Immunomodulatory Effect	Expected Immunopathogenic Shift
CD4^+^ T cells/Th1	IDO1, PGE2, TGF-β, IL-10, HGF, PD-L1/PD-1	↓ Th1 proliferation and activation; ↓ IFN-γ and other pro-inflammatory signals	Decreased Th1-mediated autoantigen-specific responses and reduced inflammation
Naïve CD4^+^ T cells	TGF-β, PGE2, IDO1, cell–cell contact	↑ generation and expansion of Treg cells	Restoration of peripheral immune tolerance
Th17 cells	PGE2/EP4, IDO1, TGF-β, IL-10, HGF	↓ Th17 differentiation and proliferation	Attenuation of inflammation
Treg cells	TGF-β, IL-10, PGE2, HGF, cell–cell contact	↑ Treg frequency and suppressive activity	Restoration of peripheral immune tolerance
CD8^+^ CTLs	IDO1, PGE2, TGF-β, PD-L1/PD-1, HLA-G	↓ proliferation and cytotoxicity	Attenuation of CTL-mediated β-cell destruction
B cells	IDO1, PGE2, PD-L1/PD-1, TGF-β, HGF, BAFF/APRIL	↓ B-cell activation and proliferation; ↓ B-cell and plasma cell survival; ↓ immunoglobulin production; ↓ autoantigen presentation by B cells	Suppressed autoantibody secretion
NK cells	IDO1, PGE2, HLA-G	↓ NK-cell proliferation, activation, cytotoxicity; ↓ IFN-γ	Attenuation of inflammation, decreased NK-cell cytotoxicity against β-cells
DCs	IL-6, IL-10, PGE2, TGF-β, IDO1	↓ DCs differentiation from monocytes and HSC; ↓ DCs maturation; ↓ MHC-II/CD80/CD86; ↓ IL-12	Reduced presentation of β-cell autoantigens
Macrophages	PGE2, IL-10, TGF-β, TSG-6, IL-1Ra, HO-1	M1 → M2 polarization; ↓ TNF-α, IL-1β, IL-6, and ROS;	Attenuation of islet inflammation and direct β-cell destruction.
Neutrophils	HO-1, PGE2, TGF-β, and other paracrine factors	↓ inflammatory activation; ↓ ROS; ↓ NETosis	Suppression of inflammation and direct β-cell damage
β-cells	HGF, TGF-β, IL-10, VEGF, TSG-6, HO-1, EVs	↓ β-cell apoptosis; ↓ inflammatory damage	Preservation of β-cell function and islet regeneration

Abbreviations: CTLs—cytotoxic T cells; DCs—dendritic cells; HGF—hepatocyte growth factor; HO-1—heme oxygenase-1; EVs—extracellular vesicles; IDO1—indoleamine 2,3-dioxygenase 1; IL-1Ra—interleukin-1Ra; IL-6—interleukin-6; IL-10—interleukin-10; PD-L1/PD-1—programmed death ligand 1 and programmed death 1 receptor; PGE2—Prostaglandin E2; ROS—Reactive Oxygen Species; TGF-β—transforming growth factor-β; TSG-6—tumor necrosis factor-stimulated gene-6; VEGF—vascular endothelial growth factor.

**Table 2:** Clinical Trials of MSC-Based Immunomodulatory Therapy in T1D.

Clinical Trial	Phase	Patients (*n*; Age; Disease Duration)	MSC	Route; Dose (×10^6^)	Follow-up (mo)	Main Outcomes	Reported Findings
NCT04078308	I/II	*n* = 20; 8–40; <6 wk	Autologous BM-MSCs	i.v. (×2); 1/kg	12	Safety; insulin; C-peptide; autoantibodies; cytokines	NR
NCT03920397	II	*n* = 7; 16–35; <4 mo	Allogeneic AD-MSCs; vitamin D	i.v.; 1/kg	6	Safety; insulin; C-peptide; HbA1c; Treg	↑ C-peptide (40%); ↓ HbA1c
NCT04061746	I/II	*n* = NR; 18–40; <6 mo	Allogeneic UC-MSCs	i.v.; 2.5/kg;	12	Safety; C-peptide; insulin; immune markers	NR
NCT03484741	I/II	*n* = 15; 18–45; NR	Autologous BM-MSCs + allogeneic UC-MSCs	i.v.; NR	6	Safety; insulin; C-peptide; HbA1c	NR
NCT00690066	II	*n* = NR; 12–35; 2–20 wk	Allogeneic BM-MSCs (PROCHYMAL^®^)	i.v. (multiple); NR	24	Insulin; C-peptide; HbA1c; GAD/IA-2 autoantibodies	NR
NCT02763423	II	*n* = 28; 12–35; <12 mo	Allogeneic UC-MSCs	i.v.; NR	24	Insulin; C-peptide; HbA1c; autoantibodies	↑ C-peptide (30%) ↓ exogenous insulin ↓ HbA1c
NCT01068951	I/II	*n* = 20; 18–40; <10 d	Autologous MSCs	i.v.; 2/kg	12	Safety; C-peptide	C-peptide preservation or increase compared with decline in controls
NCT02893306	II	*n* = 10; ≥18; <1 yr	Allogeneic BM-MSCs	i.v.; 2–3/kg	24	Safety; insulin reserve; challenge test	NR
NCT02057211	II	*n* = NR; 18–40; <3 wk	Autologous MSCs	i.v.; NR	24	C-peptide	NR
NCT01322789	I/II	*n* = NR; 12–35; <6 wk	Allogeneic BM-MSCs	i.v. (×8); NR	84	Safety; insulin; C-peptide; HbA1c; anti-GAD antibody; immune reconstitution	NR
NCT02940418	I	*n* = NR; 18–35; <3 yr	Allogeneic AD-MSCs and autologous BM-MNCs	i.v. (×2); 1 and 10/kg;	9	Safety	NR
NCT01374854	I/II	*n* = 21 + 21; 18–40; 2–20 yr	Allogeneic UC-MSCs and autologous BM-MNCs	i.a.; 1.1/kg;	96	Safety; insulin; C-peptide; HbA1c; complications	1-year follow-up: ↑ C-peptide (106%), ↓ HbA1c (12.6%), ↓ exogenous insulin (29.2%). 8-year follow-up: lower rates of neuropathy (7,1% vs. 46,7%), nephropathy (7,1% vs. 40,0%), retinopathy (7.1% vs. 33.3%), any complication (14,3% vs. 73,3%) [191,192]
NCT01219465	I/II	*n* = NR; 3–35; <9 wk	Allogeneic UC-MSCs	i.v.; 20	3	Insulin; C-peptide; HbA1c; immune reconstitution	NR
NCT06407297	I/II	*n* = 24; 8–18; <6 wk	Allogeneic UC-MSCs	i.v.; dose escalation	4	Safety; insulin; C-peptide; HbA1c; immune profile	NR
NCT01157403	I/II	*n* = NR; 10–40; <18 wk	Autologous BM-MSCs	i.v.; 2.5/kg	24	Safety; C-peptide	NR
NCT03406585	I/II	*n* = 9; 18–40; <2 yr	Allogeneic WJ-MSCs (ProTrans)	i.v.; 25/100/200	12	Safety; insulin independence; C-peptide; HbA1c	C-peptide maintained (medium or high dose administrated) [193]
NCT03973827	I/II	*n* = 10; 18–40; <3 yr	Allogeneic WJ-MSCs (ProTrans)	i.v.; 200	12	Insulin independence; C-peptide; HbA1c	↓ C-peptide 10% vs. 47% (placebo). 7-year additional follow-up: preserved 60% insulin production vs. 15% (placebo) [194]

Abbreviations: AD-MSCs—adipose-derived mesenchymal stromal cells; BM-MSCs—bone marrow-derived mesenchymal stromal cells; UC-MSCs—umbilical cord-derived mesenchymal stromal cells; WJ-MSCs—Wharton’s jelly-derived mesenchymal stromal cells; BM-MNCs—bone marrow-derived mononuclear cells; i.a.– intra-arterial; i.v.—intravenous; HbA1c—glycated hemoglobin; NR—Not Reported; d—day; mo—month; wk—week; yr—year.

## Data Availability

No new data were created or analyzed in this study.

## Abbreviations

The following abbreviations are used in this manuscript:

CTLs	cytotoxic T lymphocytes
DCs	dendritic cells
EVs	extracellular vesicles
GAD65	glutamic acid decarboxylase 65
HLA	human leukocyte antigen
HO-1	heme oxygenase-1
IA-2	insulinoma-associated protein 2
IDO	indoleamine 2,3-dioxygenase
IFN	interferon
IL	interleukin
iNOS	inducible nitric oxide synthase
MDSCs	myeloid-derived suppressor cells
MHC	major histocompatibility complex
MSCs	mesenchymal stromal cells
PGE2	prostaglandin E2
ROS	reactive oxygen species
T1D	type 1 diabetes
TGF-β	transforming growth factor-β
Th	T helper
Th0	naïve T helper
Th1	T helper 1
Th17	T helper 17
TNF-α	tumor necrosis factor alpha
tolDCs	tolerogenic dendritic cells
Tregs	regulatory T cells
VEGF	vascular endothelial growth factor
ZnT8	zinc transporter 8
